# Innovations and Inventions: Why Was the Ugi Reaction Discovered Only 37 Years after the Passerini Reaction?

**DOI:** 10.1021/acs.joc.2c00792

**Published:** 2022-07-26

**Authors:** Alexander Dömling

**Affiliations:** Department of Drug Design, University of Groningen, Groningen 9700 AD, The Netherlands

## Abstract

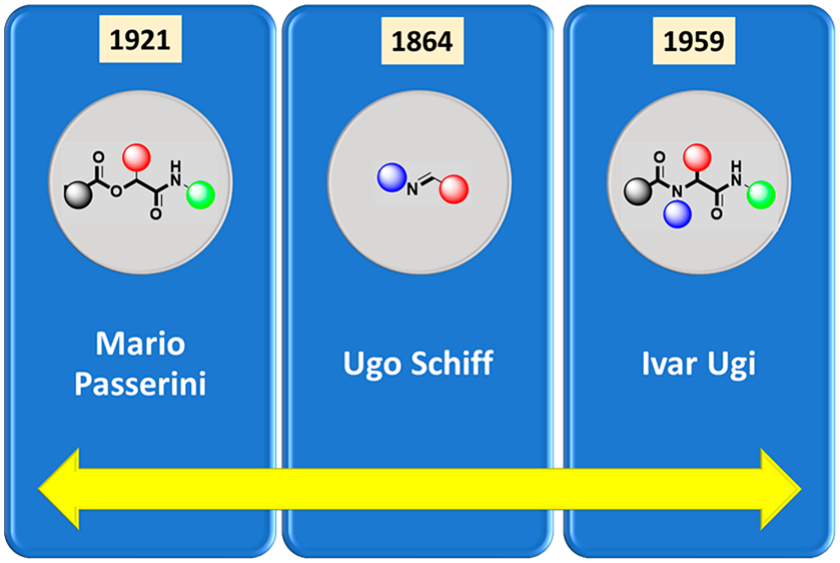

This year represents the 100th anniversary of the discovery of the Passerini three-component reaction. The related Ugi four-compound reaction was discovered 37 years after the Passerini reaction. Undoubtedly, both reactions are very important multicomponent reactions but the Ugi reactions outperform the Passerini reactions in terms of combinatorial space according to the equation *x^y^* [*x* is the number of building blocks per component, and *y* is the order of the multicomponent reaction (for Passerini, *y* = 3; for Ugi, *y* = 4)]. In this work, a historical but contemporary perspective of the discoveries and innovations of the two reactions is given. From a bird’s eye view and in a more general sense, the discovery of novel reactions is discussed and how it relates to inventions and innovations.

## A Historical Perspective

**Figure 1:**
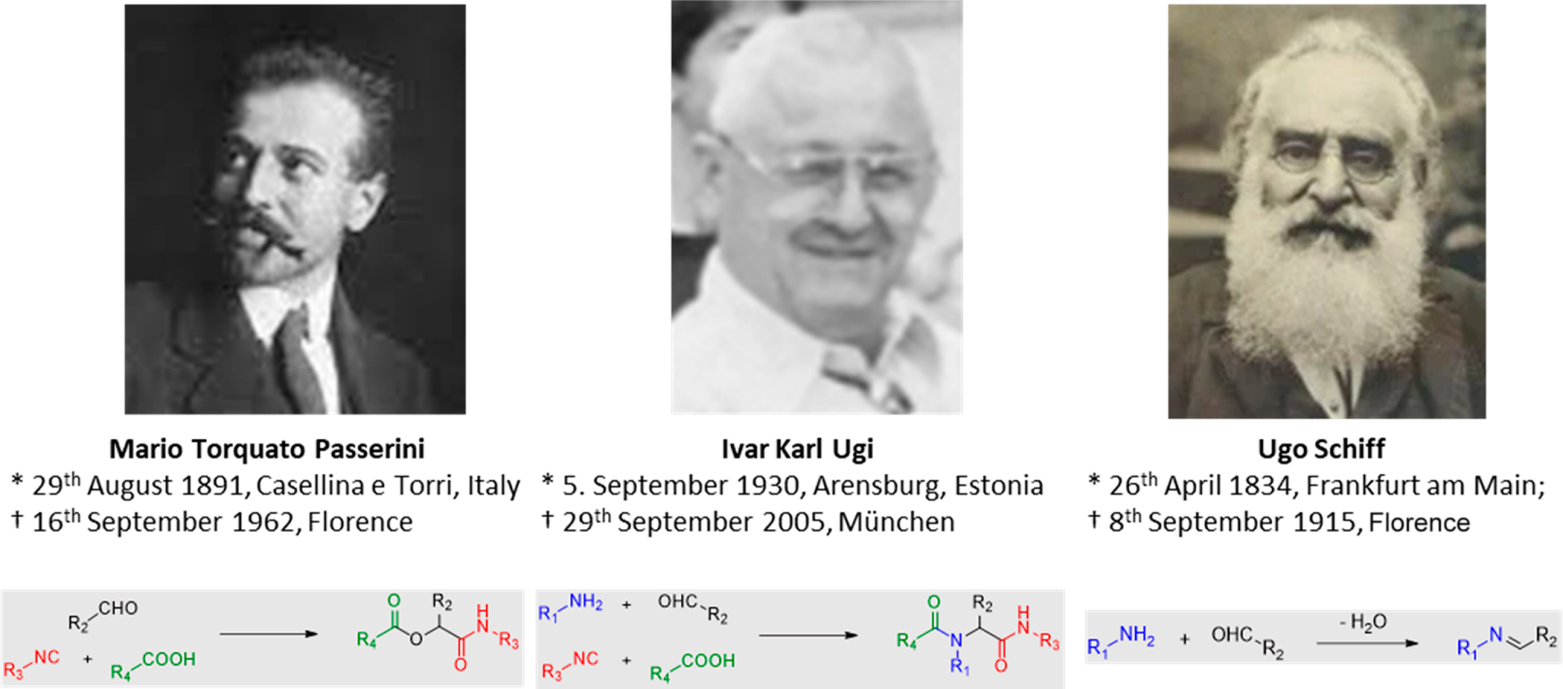
Portraits of the key figures of isocyanide-based multicomponent reactions (IMCRs) and Hugo Schiff and their corresponding reactions. The pictures of Passerini and Ugi are courtesy of Sara Passerin and Dr. Konstantina Kehagia, respectively.

Both the Passerini three-component reaction and the Ugi four-component reactions are very important tools in modern organic chemistry with plenty of synthetic and industrial high-value applications.^1^ They are named after their inventors, Mario Passerini (1891–1962) and Ivar Karl Ugi (1930–2005), respectively (Figure 1). Passerini was born in Casellina e Torri (now a neighborhood of Scandicci, Italy) on August 29, 1891. He graduated from the University of Florence in 1916 in chemistry and pharmacy. He interrupted his university studies to participate in the first world war, received the cross of war merit, and was discharged with the rank of lieutenant for war merits. In 1924, Mario Passerini obtained the “venia legendi” and became professor for pharmaceutical chemistry at the University of Siena. He moved back to Florence in 1933, where he became Ordinarius of Pharmaceutical Chemistry. He first published his famous reaction in 1921.^2^

Ivar Karl Ugi was born on the island of Sareema, Estonia, and his family fled to Germany during the second world war, when the Soviets invaded Estonia. He studied chemistry and mathematics at the University of Tübingen until 1951 and finished his Ph.D. in 1954 with Rolf Huisgen.^3^ He performed his habilitation in 1960 on “Isonitrile und Pentazole” and could prove for the first time the existence of the very unstable pentazole ring system by crystallization and other means.^4^

In the course of his studies, he was interested in the isosteric tetrazoles and its synthesis, to compare the analytical and chemical similarities and differences of the two heterocycles. A well-known synthetic access to tetrazoles was the [3+2]-cycloadditions of hydrazoic acid to isocyanides.^5^ This is likely the way Ugi came to possess several isocyanide derivatives, otherwise a rather fancy not every day functional group.^6^ From 1962 to 1968, he worked for Bayer Co. and rapidly became Forschungsdirektor des Hauptlaboratorium (“CSO”) in Leverkusen. From 1968 on he was professor at the University of Southern California in Los Angeles, and in 1971, he accepted the Lehrstuhl für Organische Chemie I at the Technische Universität München, as the successor of Friedrich Weygand. In addition to isocyanide-based multicomponent reaction (MCR) chemistry, his primary contributions included computational retrosynthesis and group theory (theory of BE- and R-matrices),^7^ rearrangement and stereochemistry of pentacoordinated phosphorus,^8^ and ferrocene chemistry, which has led to the development of the industrially important class of chiral ligands Josiphos and Taniaphos derived from the enantiopure (*R*)-*N*,*N*-dimethyl-1-ferrocenylethylamine (“Ugi’s amine”).^9^ His first publication of the Ugi multicomponent reaction “Versuche mit Isonitrilen” was published in 1959,^10^ and in just a handful of years, he described all of the major variants of his versatile MCR.^11^

Interestingly, another chemist, Ugo (Hugo) Joseph Schiff (1834–1915), considerably overlapped with Passerini at the University of Florence (Figure 1).^12^ In fact, Passerini was Schiff’s student. Borne in Göttingen, Germany, Schiff was scientifically raised by Friedrich Wöhler. In 1863 he went to Italy, working in Pisa on the condensation reaction of aldehydes and primary amines.^13^ The products are called imines or, to his honor, Schiff bases and are very important intermediates in many organic reactions. Schiff bases and aldehydes and ketones have an often-similar carbonyl-type reactivity. Schiff then remained in Florence for his entire career, which lasted 50 years (from 1864 until 1915). It is interesting to note the connection between Hugo Schiff and other MCR inventors. Mario Betti (1875–1942) was a student of Schiff and developed the reaction in 1900, while working in Schiff’s laboratories. Pietro Biginelli (1860−1937) graduated in Torino and then moved to Milano; however, from 1890 to 1897, he was in Florence, in Schiff’s laboratories, and it was there that he discovered, in 1893, the famous multicomponent reaction.

**Figure 2:**
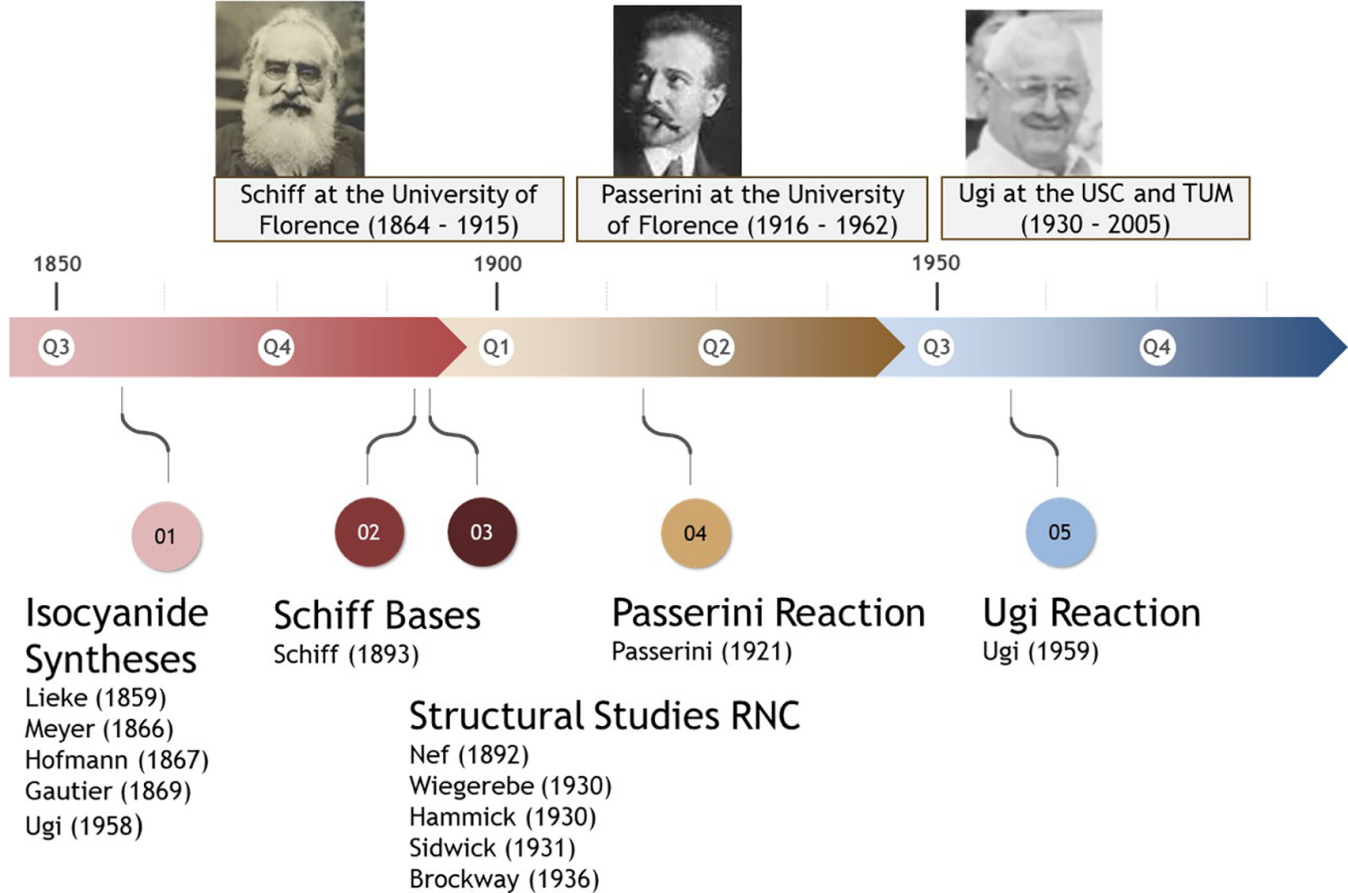
Important milestones of isocyanides and isocyanide-based multicomponent reactions of Passerini and Ugi and the spatiotemporal overlap of the two chemists, Passerini and Schiff, at the University of Florence. The pictures of Passerini and Ugi are courtesy of Sara Passerini and Dr. Konstantina Kehagia, respectively.

In mechanistic terms, one could argue that the Passerini and Ugi reactions are quite similar and that the Ugi four-component reaction is just a combination of the Passerini three-component reaction with the Schiff reaction. Due to the superficial similarity of the two reactions and considerable spatiotemporal overlap of the two professors, Passerini and Schiff, both at the University of Florence, one could ask the provoking question of why the Ugi reaction was discovered only 37 years after the Passerini reaction (Figure 2).

## Invention

The invention of new reactions is exceptionally important to synthetic organic chemistry and is a worthwhile occupation. While there are only a few elementary steps, the combination of elementary steps in a defined way, giving rise to a specific class of products with a certain scope regarding the electronic and steric nature of the starting materials, is often defined as a reaction in organic chemistry. For example, the Wittig reaction can be formulated involving ylide formation, followed by an irreversible [2+2]-cycloaddition to give an oxaphosphetane intermediate, and decomposition of the four-membered ring comprising both a Berry pseudorotation process and P–C and C–O bond breakage, stereospecifically releasing the alkene product.

How are new reactions discovered? The historical approach to discovering new reactions involves human creativity and/or serendipity. “Serendipity” implies the finding of one thing while looking for something else. The role of serendipity in scientific discoveries is well established. However, only a good scientist is able to analyze the discovery and to recognize the potential of such coincidences in the first place. As the French chemist Luis Pasteur phrased it “Dans les champs de l’observation, le hasard ne favorise que les esprits préparés”. The exact ratio of chance to knowledge and creativity in the history of the discovery of the two multicomponent reactions is unknown. Another approach to new reactions is based on rational design, e.g., analogy thinking. For example, the finding that thiocarboxylic acids react highly regioselectively in the Ugi reaction, combined with a specific multireactive isocyanide, Schöllkopf’s isocyanide, yielded a useful reaction leading to highly substituted thiazoles in one pot.^14^ Yet another modern approach is using artificial intelligence. Ironically, Ivar Ugi was also a trailblazer of computational chemistry and developed pioneering programs for analyzing relationships between educts and imaginary products, hence computational retrosynthesis.^15^ This way the user could predict and experimentally validate new reactions. An analytical approach to new reactions used the systematic mixing and analysis of multicomponent mixtures. The prediction and execution of new reactions and synthesis pathways to produce valuable compounds is a topic of outstanding importance in current and future chemical engineering research. For example, the automated, miniaturized nanosynthesis in combination with HT screening was recently shown to efficiently screen many different catalytic conditions for C–N coupling across a wide range of complex substrates (‘high-throughput experimentation’).^16^

## Innovation

**Figure 3:**
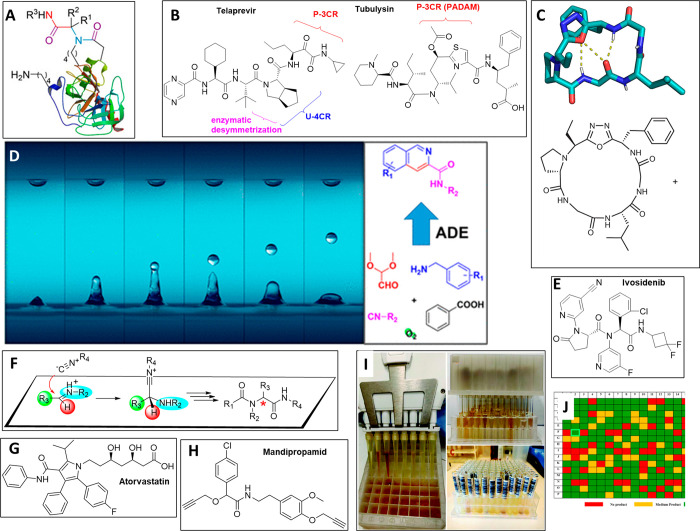
Examples of innovative applications of the Passerini and Ugi reactions. (A) Highly site-selective protein modification reacting surface Lys and Asp/Glu in an Ugi reaction. (B) Telaprevir and tubulysin both accessed by IMCR. (C) Two-dimensional and crystal structure of a peptide macrocycle with an oxadiazole backbone graft (synthesized by Ugi reaction) for enhancing membrane permeability (CCDC 1497735). (D) Novel isoquinoline synthesis based on a key Ugi reaction step performed in a highly miniaturized manner in 2.5 nL droplets employing an acoustic droplet ejection platform. (E) First-in-class anticancer drug ivosidenib derived by Ugi 4CR. (F) Key stereogenic step of the Ugi reaction, in which the isocyanide adds to the prochiral Schiff base to form the chiral nitrilium ion, which undergoes further steps to afford the final Ugi product. (G) Cardiovascular disease blockbuster drug atorvastatin can be non-obviously synthesized by Ugi 4CR. (H) Fungizide mandipropamid produced by Passerini 3CR. (I) Ninety-six-well parallel synthesis of high-quality isocyanides on a millimole scale stored in barcoded vials. (J) MS analysis of the acoustic droplet ejection-enabled automated reaction scouting of a novel isoquinoline MCR in a 384-well format.

How can the innovative aspects of the two multicomponent reactions be described? The invention of the original reactions together with expansion of the scope in myriad additional works paves the way for innovative applications of the two reactions. Some recent by no means comprehensive examples are chosen to underscore this (Figure 3).

Bioconjugation methods employing MCR chemistry make up a particularly promising field of research^17^ and have been applied to innovations in glycopeptides,^18^ lipopeptides,^19^ vaccines,^20^ cyclic peptides (Figure 3C),^21^ macrocycles,^22^ biolabeling,^23^ and stapled peptides.^24^ A particularly innovative application of Ugi MCRs encompasses the site-selective modification of proteins (Figure 3A).^25^ Due to the redundancy of surface amino acid side chains on the surface of larger proteins, monoreactive modifications often yield mixtures of mono-, di-, tri-, tetra-, etc., substitutions and yield Gauss-type product distributions. The Ugi 4CR involving proteins has been realized by using exposed amino acid side chains -COOH (Asp and Glu) and -NH_2_ (Lys) to conjugate two more components, isocyanide and the oxo component. Different antibody conjugates were synthesized with the model mAb tratuzumab.

The drug telaprevir has received FDA approval for the treatment of hepatitis C and is a reversible inhibitor of the HCV serine protease. A highly convergent stereospecific and scalable synthesis has been described on the basis of the combination of the Passerini and Ugi MCR, combined with biocatalytical desymmetrization to produce the central bicyclic Schiff base (Figure 3B).^26^ The highly innovative and scalable MCR route outperforms the commercial route by 10 steps (approximately one-third of the total length) with, overall, much improved yields. Clearly, a similar approach can be envisioned for the SARS-CoV-2 3CLpro-targeting nirmatrelvir. Tubulysin, a myxobacterial nonribosomal tetrapeptide, is a highly cytotoxic compound of interest in antibody drug conjugates. The fermentative production has so far not been determined, and all tubulysin for commercial purposes is produced by total syntheses. The Passerini 3CR was used as a key step to assemble the tubulysin skeleton in fewer than 20 steps in overall ∼30% yield, resulting in one of the shortest and most scalable syntheses of this complex natural product (Figure 3B).^27^ Several other short and convergent routes using MCRs have been described.

DNA-encoded combinatorial synthesis is a successful technique in early drug discovery in which minute amounts of very large numbers of compounds are produced and screened. The Ugi reaction and several variations of the Ugi reaction are well-tolerated by DNA on the solid phase and show a broad scope.^28^

A major difference between the Ugi and Passerini reactions is based on their mechanisms, which are polar and nonpolar, respectively, and consequently their solvent preference, polar protic and nonpolar. This has major implications for the development of catalysts to produce chiral products (Figure 3F).^29^ While the enantioselective Passerini reaction has been partially solved, the Ugi reaction is still awaiting a satisfactory solution.^30^ The enantioselective Ugi reaction was described for an atypical aprotic apolar solvent, also involving a chiral ligand that is very complex to synthesize, which restricts its utility.

The Ugi MCR found exciting applications in information technology. Using the Ugi 4CR, more than 1.8 million bits of art historical images were encoded and decoded by mass spectrometry, including a drawing by Picasso.^31^ Securing communication channels via message encoding is another hot topic in information technology. Specifically designed molecular keys were introduced by combining advanced encryption standard cryptography with molecular steganography based on the Ugi 4CR. The necessary molecular keys require great structural diversity, suggesting the application of multicomponent reactions.^32^ The Ugi 4CR of perfluorinated acids was utilized to establish an exemplary database consisting of 130 commercially available building blocks. Considering all permutations, this combinatorial approach can unambiguously provide 500 000 molecular keys in only one synthetic step per key. The molecular keys are transferred nondigitally and concealed by adsorption onto either paper, coffee, tea, or sugar and by dissolution in a perfume or in blood. Re-isolation and purification are accomplished by the perfluorinated side chains of the molecular keys. High-resolution tandem mass spectrometry can unequivocally determine the molecular structure and thus the identity of the key for a subsequent decryption of an encoded message.

While many approved drugs can be synthesized using isocyanide-based MCRs, only a few were originally discovered using MCR. Ivosidenib is an isocitrate dehydrogenase-1 (IDH1) inhibitor and was recently approved for the treatment of acute myeloid leukemia (AML). Ivosidenib is clearly based on the α-aminoacylamide scaffold of the Ugi 4CR and was discovered by this route and is also produced by the same reaction (Figure 3E).^32^ MCRs can also find widespread applications in the advantageous synthesis of generics.^33^ For example, the previously best-selling drug atorvastatin can be assembled by a central Ugi reaction in an overall short and high-yield synthesis that outperforms the original Paal–Knorr route (Figure 3G).^34^ Mandipropamid, a fungicide that targets the cellulose synthase and inhibits cell wall biosynthesis in the oomycete plant pathogen *Phytophthora infestans*, has widespread applications in grape, potato, tomato, and cucurbit protection.^35^ The commercial compound can be made by a sequence involving the Passerini reaction as a key step, where this isocyanide is formed *in situ* (Figure 3H).^36^

Multicomponent reaction chemistry performed at the nanoscale in an automated fashion was recently introduced using acoustic droplet ejection (Figure 3D). It can be applied for the synthesis of boronic acid, indole, isoquinoline, electrophiles, α-hydroxyacylamide (Passerini scaffold), and other compound libraries, in a highly sustainable synthesis approach (Figure 3J).^37^

Isocyanide-based MCR experienced a renaissance in polymer chemistry for the synthesis of sequence-defined polymers, engineered organic porous networks, and polymers for biomedical applications, data storage, etc.^38^

Many applications mentioned above need building block diversity not only in the oxo, amino, and acid components but also in the isocyanide component. However, the limited access to isocyanides hampers progress in IMCR. Many novel approaches to specific convertible isocyanides that can be further functionalized and provide interesting chemistries have been taken. Other approaches avoid isocyanide isolation and employ *in situ* preparation and usage. Broad, experimentally easy, and sustainable access to hundreds of high-quality isocyanides in a parallel approach (based on a 96-well format) was recently demonstrated (Figure 3I).^39^ The key to this procedure is the dry workup by simple silica filtration. This way many unprecedented isocyanides thought to be unstable could be produced, and their novel chemistries can now be investigated.

Novel chemical reactions, when elaborated with a great scope, often give rise to innovation and can have a major impact on society. Examples include the Ziegler–Natta polymerization to polypropylene and the olefin metathesis reaction used in the Shell higher-olefin process and for the production of numerous pharmaceutical ingredients. MCRs differ from classical one- or two-component reactions by the substrate multiplicity. Many features of these two MCRs are related to their unique multicomponent nature. The construction of complex functional molecules in one step or a few steps is uniquely possible according to the principle “form follows function”. Properties can be more efficiently optimized. MCRs are highly sustainable reactions often working under environmentally benign conditions and more importantly avoiding waste and/or byproducts by reducing the number of steps. Moreover, the compatibility of the Passerini and Ugi reactions with most orthogonal functional groups allows for virtually endless combinations with other reactions to span a truly accessible very diverse scaffold space.^40,41^ Arguably, the Ugi and Passerini MCRs belong to the same class of highly productive, transformative synthetic chemistry reactions that give innovators unique tools to discover and produce novel chemical solutions for the problems of the exponentially growing world population and related scaling issues of growing globalization. The rhetorical question of the title, ‘Why Was the Ugi Reaction Discovered Only 37 Years after the Passerini Reaction?’, cannot be conclusively answered on the basis of historic facts and without speculation and is left to the reader’s fantasy.

## Dedication

Dedicated to the great chemists Ivar Ugi and Mario Passerini.
